# Effects of competitive pressure and habitat heterogeneity on niche partitioning between Arctic and boreal congeners

**DOI:** 10.1038/s41598-021-01506-w

**Published:** 2021-11-11

**Authors:** Anne-Sophie Bonnet-Lebrun, Thomas Larsen, Morten Frederiksen, Derren Fox, Fabrice le Bouard, Aude Boutet, Þorkell Lindberg Þórarinsson, Yann Kolbeinsson, Tanguy Deville, Norman Ratcliffe

**Affiliations:** 1 British Antarctic Survey, Natural Environment Research Council, High Cross, Madingley Road, Cambridge, CB3 0ET UK; 2 Department of Archaeology, Max Planck Institute for the Science of Human History, Kahlaische Strasse 10, 07745 Jena, Germany; 3 Department of Bioscience, Aarhus University, Frederiksborgvej 399, 4000 Roskilde, Denmark; 4 Northeast Iceland Nature Research Centre, Hafnarstétt 3, 640 Húsavík, Iceland

**Keywords:** Animal behaviour, Behavioural ecology, Climate-change ecology

## Abstract

The rapidly changing climate in the Arctic is expected to have a major impact on the foraging ecology of seabirds, owing to changes in the distribution and abundance of their prey but also that of competitors (e.g. southerly species expanding their range into the Arctic). Species can respond to interspecific competition by segregating along different niche axes. Here, we studied spatial, temporal and habitat segregation between two closely related seabird species: common guillemot *Uria aalge* (a temperate species) and Brünnich’s guillemot *Uria lomvia* (a true Arctic species), at two sympatric sites in Iceland that differ in their total population sizes and the availability of marine habitats. We deployed GPS and temperature-depth recorders to describe foraging locations and behaviour of incubating and chick-rearing adults. We found similar evidence of spatial segregation at the two sites (i.e. independent of population sizes), although segregation in environmental space was only evident at the site with a strong habitat gradient. Unexpectedly, temporal (and, to a limited extent, vertical) segregation appeared only at the least populated site. Overall, our results show complex relationships between the levels of inferred competition and that of segregation.

## Introduction

A species’ *fundamental niche* is broadly constrained by a set of environmental conditions^1^. However, the species’ *realised niche* is usually smaller due to factors such as dispersal limitation and biotic interactions. Interspecific competition is one such biotic factor, and its potential role in refining species ranges has been highlighted in various taxa^2,3^. At their range limits, closely related species often co-occur in areas of sympatry. There, competition pressure should be strong when shared resources become limiting, because of increased population density^4^ and/or an environmentally-driven decline in resource availability^5^.

Species can coexist in areas of sympatry even when sharing the same resource by segregating temporally or spatially^6^. Spatial segregation can be horizontal^7,8^ or vertical^2,9^. It is often structured by habitat heterogeneity, as subordinate species can be displaced from otherwise suitable habitats when their dominant competitor is present^10,11^. In these cases, the ability of subordinate species to escape competition can depend on the availability of refugia habitats that are avoided by dominants^2,6,12^.

Many species respond to climate change by shifting their ranges latitudinally or altitudinally^13^. Not all species are able to shift their range to the same degree (e.g. polar or montane species cannot shift their ranges any further^14,15^). This will likely modify species interactions at the margins of species ranges, increasing competitive pressure in areas of sympatry, with species at the trailing edge of their range potentially more likely to be subordinate^16,17^ outside of refugia. For example, in the Arctic, where rates of warming are among the most rapid on Earth^18^, the northward shift of red foxes *Vulpes vulpes* increases competitive pressures on Arctic foxes *Alopex lagopus*^17^.

This is true in terrestrial environments, but also in marine ecosystems. In particular, the Atlantic sector of the Arctic is affected by increased inflow of warm, saline waters from the south into Arctic latitudes^19^, leading to a borealisation of copepod and fish communities^20^. Similarly, increases in boreal seabird abundance^21^ might increase competitive pressure on already declining Arctic species^22^.

Among seabirds, two species provide a classic example of competitive interactions in the Arctic: common (CG) and Brünnich’s guillemots (BG) are sister species occupying temperate and Arctic latitudes, respectively. Their ranges overlap in the sub-Arctic (Irons et al. 2008), Iceland being the most important zone of sympatry in the Atlantic^23^. BG are declining in parts of their Atlantic range^22–24^ and CG may be shifting northward in some areas^25^. CG are dominant over BG at multiple sites within their zones of sympatry when competing for nest sites^26^ and food^5^. A northward shift of CG could therefore potentially elevate competitive pressure on BG.

To study the factors affecting niche partitioning in CG and BG we tracked incubating and chick-rearing BG and CG using GPS and Temperature-Depth Recorders (TDR) from two colonies in northern Iceland with contrasting levels of competition and habitat accessibility. We investigated: (1) spatial (horizontal and vertical) segregation, (2) temporal partitioning in foraging activity and (3) whether spatial segregation was underpinned by different habitat uses. We contrasted results between colonies, hypothesizing that segregation would increase in relation to competitive pressure and habitat heterogeneity.

## Methods

### Data collection

#### Study sites

Látrabjarg (24.52° W; 65.50° N) is located at the westernmost tip of Iceland and, in 2007–08, hosted ca. 343,900 guillemot pairs (ratio of CG:BG = 1.9:1) on enormous mainland sea cliffs^23^. It is influenced by the warm and saline Irminger current flowing clockwise around the Icelandic coast, and the cold East Greenland Current flowing southwards along the Greenland coast. Grímsey (18.02° W; 66.57° N) is a small island off the north of Iceland in the warm Irminger current flowing from the south-west, and within foraging distance of the cool East Icelandic Current flowing from the north-west. It hosts ca. 71,400 guillemot pairs (ratio of CG:BG = 16.4:1)^23^. The two sites are 360 km apart by the shortest sea journey.

#### Tracking

111 combinations of Pathtrack nanoFix GPS loggers and Cefas G5 TDR loggers were deployed on 63 CG *Uria aalge* (38 at Látrabjarg and 25 at Grímsey) and 48 BG *Uria lomvia* (35 at Látrabjarg and 13 at Grímsey) between 11 June and 4 July 2019 (late incubation and chick rearing). GPS devices recorded locations every 3 min, and TDR devices recorded depth and temperature every second. For more details, see Supporting Information. All animals in this study were handled in accordance with^27^. The experimental protocols were approved by the British Antarctic Survey Animal Welfare & Ethical Review Board.

### Data analysis

#### Processing of TDR data

TDR data were analysed using the *diveMove* package^28^ in R (R Core Team 2019), subsampling data to every 3 s to save computing power. To remove artefacts due to temporal changes in accuracy of pressure transducers, a zero-offset correction was applied, following^29^ with a diving threshold of 3 m. The package was used to identify dives and estimate their start time and maximum depth. Based on the TDR time series, time was categorised into four activities for each bird (at the colony, flying, on the sea surface and diving) (see Supporting Information). The few sections of TDR data indicating isolated or prolonged records > 200 m were removed, as well as ‘dives’ corresponding to times when the birds were at the colony, which were likely due to the birds pressing the transducer when preening.

#### Processing of GPS data

Unreliable GPS locations were defined by visual inspection and device Accuracy Indicator < 30. After removing these locations, trips were separated manually based on when birds left/returned to the colony. Since the TDR and GPS sampling rates were asynchronous, the locations of birds at the start time of each dive were predicted using linear interpolation on projected GPS data (using an Equal-Area Scalable Earth Grid; ESPG: 3408).

#### Habitat use

Habitat was characterised at each diving location along several axes: sea surface temperature (SST), bathymetry, inside vs. outside a fjord, and, at Látrabjarg, distance from the ice edge. SST was estimated using TDR temperature data during periods when the bird was on the sea surface (see Supporting Information). TDR data was used in preference to satellite-derived SST to produce measures at the exact times and locations of dives.

Bathymetry was obtained using the GEBCO depth raster (resolution: 30’’; https://www.gebco.net/), extracting depth at the location of each dive. At Látrabjarg, the only study colony from which the Marginal Ice Zone (MIZ) is accessible, the distance between each diving location and the ice edge the previous day (downloaded as a daily shapefile at https://www.natice.noaa.gov/products/kml_daily_arc.html) was also calculated. Last, whether locations fell within a fjord or not was quantified by manually drawing polygons around Icelandic fjords on QGIS^30^ and rasterising the obtained shapefile (resolution: 5 km).

#### Niche overlap

Overlap in diving depths and times, and habitat characteristics (temperature and bathymetry) was quantified following Geange et al. (2011), using the formula:

$$O_{i,j}^{s} = 1 - \frac{1}{2}*\smallint \left| {f_{i}^{s} \left( x \right) - f_{j}^{s} \left( x \right)} \right|dx$$

where x is the variable and $f_{i}^{s}$ and $f_{j}^{s}$ are utilisation distributions (UD) for species *i* and *j* along the variable axis at site *s*. UDs were estimated using a kernel density of the frequency distributions of each variable (at each dive location) for each species and site, using the *density* function from the *stats* R package and a common bandwidth (see Table 1).

**Table 1:** Overlap between common and Brünnich’s guillemots along different axes.

	Bandwidth	Grímsey all stages		Látrabjarg all stages		Látrabjarg incubation		Látrabjarg chick rearing	
		Obs	*p*-value	Obs	*p*-value	Obs	*p*-value	Obs	*p*-value
Horizontal	7.5 km	0.41	**0.004**	0.36	** < 0.001**	0.22	** < 0.001**	0.55	0.369
Temporal	1 h	0.60	**0.024**	0.89	0.340	0.91	0.690	0.84	0.370
Vertical	5 m	0.68	0.066	0.92	0.550	0.85	0.511	0.87	0.187
Sea surface temperature	1 °C	0.85	**0.020**	0.77	**0.008**	0.74	0.109	0.80	0.120
Bathymetry	50 m	0.57	**0.017**	0.80	0.112	0.60	**0.010**	0.81	0.355

“Obs.” = observed segregation value; “*p*-value” = proportion of simulated overlap values (from species labels permutations) that are lower than the observed. p-values below 0.05 are highlighted in bold.

To compare foraging areas, kernels of space use (spatial UDs) were calculated for each species and colony separately, with a common bandwidth of 7.5 km (based on prior exploration for each colony and species separately, using the reference bandwidth selection method). Overlap in foraging areas between species was calculated with the UD Overlap Index (UDOI^31^, using the *kerneloverlap* function in the *adehabitatHR* R package^32^.

Whether the two species overlapped less than by chance, as well as whether the use of the ice edge at Látrabjarg was similar between species, was tested following^33^, i.e. using a permutation approach (see Supporting Information). Overlap values were compared between sites or breeding stages using a bootstrap procedure (see Supporting Information).

In addition to segregation indices, to test whether the characteristics of the habitat the two species share differed from that they use exclusively, the SST recorded for diving periods that were within the area of overlap between CG and BG UDs (“$CG \cap BG$”) was compared with those sections of each species’ UDs that were used exclusively (“$CG\backslash BG$” and “$BG\backslash CG$”).

At Látrabjarg, breeding stages (incubation vs. chick rearing) were also investigated separately. At Grímsey, only one BG was tracked during incubation (see Fig. S1 for sample sizes) and for both species combined, there was no evidence of stage-specific foraging (Fig. S2) so data were pooled.

## Results

### Foraging behaviour and spatio-temporal overlap

**Figure 1:**
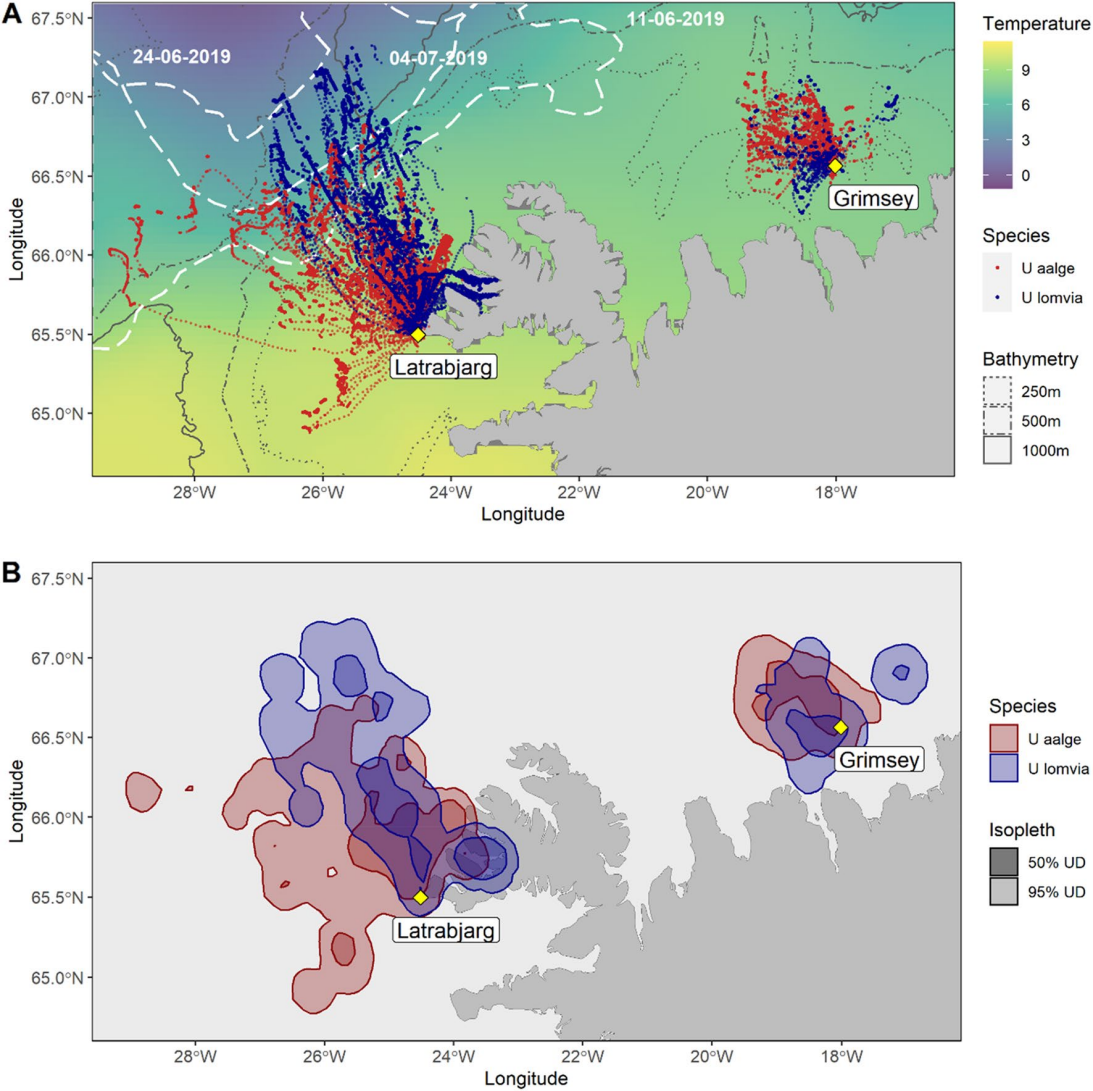
(**A**) Map of tracking data (small dots) and predicted dive locations (large dots), separated by species (red: common guillemot, *U. aalge;* blue: Brünnich’s guillemot, *U. lomvia*). White lines: external limit of the Marginal Ice Zone at the start, middle and end of the study. Sea surface temperature extracted from https://podaac-opendap.jpl.nasa.gov:443/opendap/allData/ghrsst/data/GDS2/L4/GLOB/JPL/MUR/v4.1 and averaged across the study period. (**B**) At-sea utilisation distributions (UD) and their overlap. Red: common guillemots 50% UD (dark) and 95% UD (pale); blue: Brünnich’s 50% UD (dark) and 95% UD (pale). Bandwidth for kernel calculations: 7.5 km. Yellow diamonds and labels: colony locations and names. Maps generated in R, version 4.1.1. (https://www.R-project.org/).

We recovered 52 GPS and 52 TDR from CG (83%), and 38 GPS and 40 TDR from BG (79 and 83%; Fig. S1 and Table S2). One TDR logger malfunctioned, and as only tracks with both GPS and TDR data available were used, in total 50 CG and 38 BG were available for analysis (Table S2). Both species at Látrabjarg performed longer foraging trips and individual flying sections than birds from Grímsey (Fig. 1, Tables S3, S4 and S5). Consequently, birds from Látrabjarg covered an at-sea area 3.4 times larger than at Grímsey (area inside the union of the 95% UD of the two species: 27,840 km^2^ at Látrabjarg, 8,257 km^2^ at Grímsey). Within sites, the duration of foraging trips and flying sections did not differ significantly between species (Tables S3, S4 and S5).

Interspecific horizontal overlap was similar across sites (Fig. 3). At Látrabjarg, horizontal overlap was lower than expected by chance during incubation but not chick rearing (Table 1, Figs. S3, S4).

**Figure 2:**
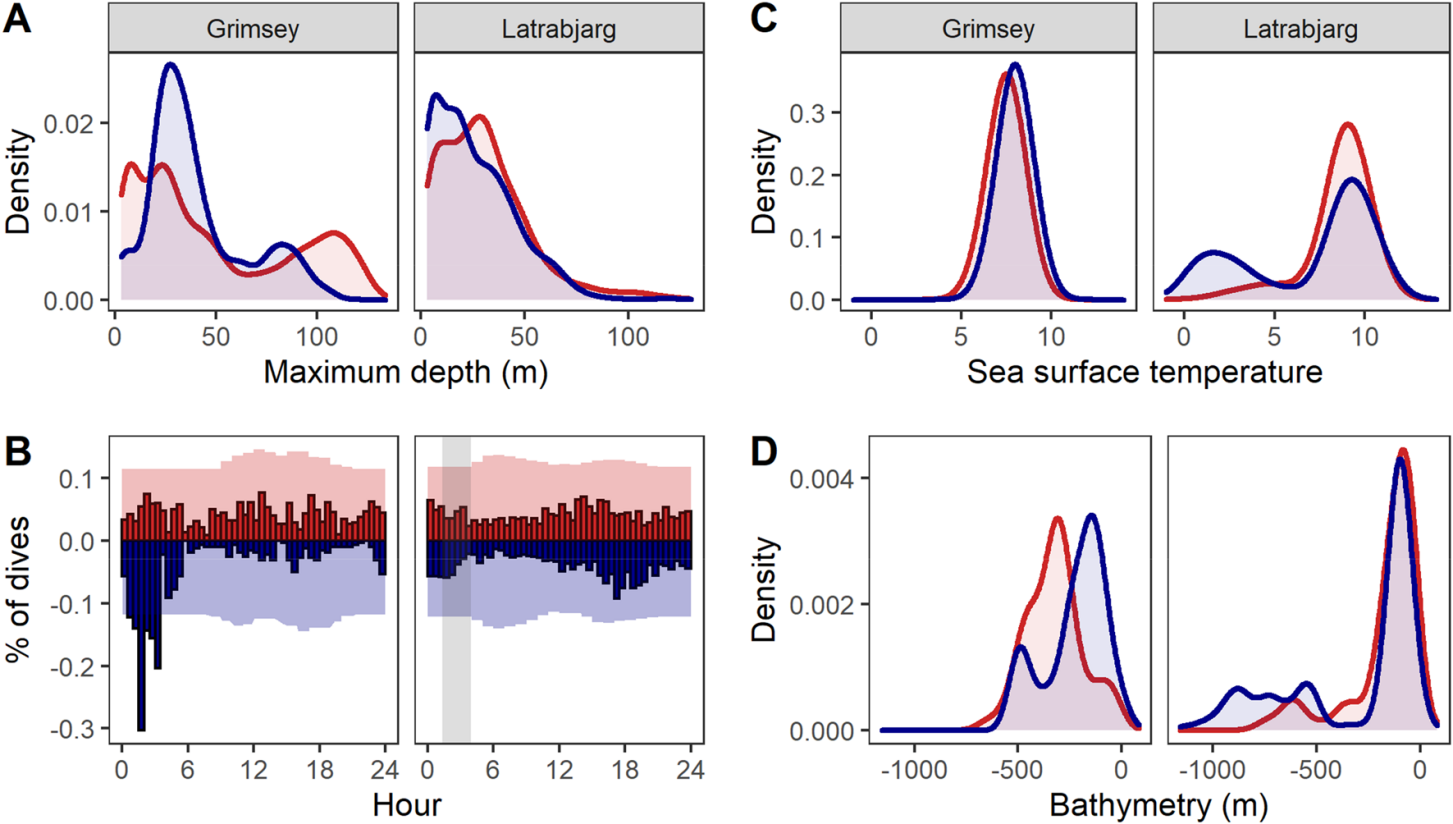
Diving behaviour and conditions at dives across colonies and species (red: common guillemot; blue: Brünnich’s guillemot). (**A**) Distribution of maximum dive depth; (**B**) Diel distribution of the occurrence of dives; shaded areas: relative distribution of tracking effort (hours in GMT); grey areas: times between sunset and sunrise (averaged over all deployment dates for each site). (**C**) Sea surface temperature (°C); (**D**) Bathymetry (m). Values averaged across diving sections (series of dives separated by flight).

**Figure 3:**
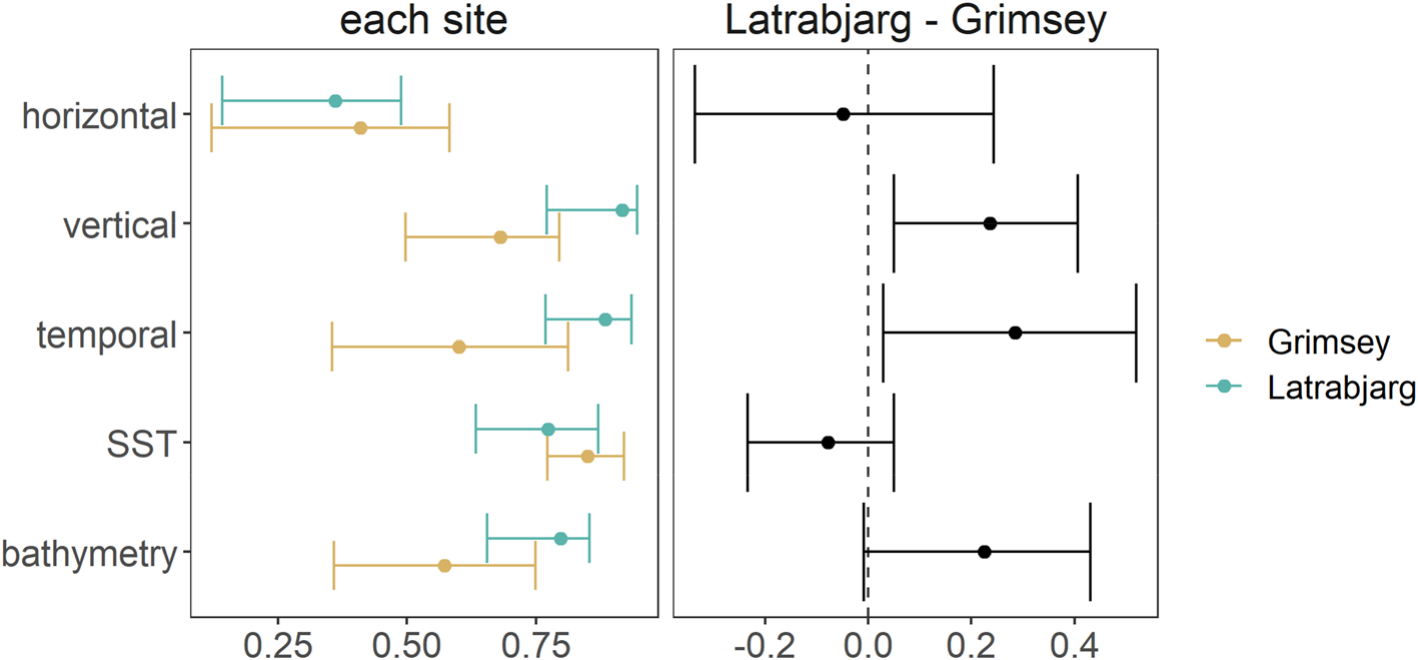
Overlap values at each site (left) and difference between Látrabjarg and Grímsey (right): observed values and bootstrap confidence intervals. Positive values in the right panel correspond to higher overlap at Látrabjarg than at Grímsey (i.e. more segregation at Grímsey). SST = Sea Surface Temperature.

At Grímsey, both species dived to shallow/intermediate depths, but also to deep depths, and the second peak in diving depths was more marked and deeper for CG (below 100 m) than BG (~ 80 m, Fig. 2A). Nevertheless, the permutation test of overlap was marginally above the α = 0.05 threshold of significance (Table 1). CG dived throughout the day, while BG did so more around the darkest hours of the day (Fig. 2B), resulting in lower temporal overlap than expected by chance (Table 1). In contrast, at Látrabjarg, both species dived to shallow or intermediate depths (mostly above 50 m, Fig. 2A) throughout the day during both breeding stages (Fig. S5). Vertical and temporal interspecific overlaps were therefore lower at Grímsey than at Látrabjarg (Fig. 3).

### Habitat use and niche overlap

At both sites, overlap in SST was lower than expected by chance (Table 1). At Látrabjarg, both species dived in warm waters (~ 9 °C, Fig. 3A), but BG also dived in cold waters (0–5 °C, Fig. 2C), driving the apparently lower overlap observed at this site (Table 1), although the site difference was not significant (Fig. 3). At Grímsey, BG foraged mostly in waters ~ 200 m deep, and CG foraged over variable bathymetries, mostly between 0 and 600 m (Fig. 2D). At Látrabjarg, both species foraged in waters ~ 200 m deep, but also performed a limited number of dives in deeper waters (~ 600 to ~ 1200 m). Overlap in bathymetry use was lower than expected by chance only at Grímsey (Table 1).

At Látrabjarg, where sea ice was accessible to the birds, BG regularly dived along or in the MIZ, whereas CG rarely did so (Fig. S6). The difference between species in percentage of dives < 5 km from the ice edge was 22.9%, which was higher than expected by chance (p-value from the permutation test < 0.001). This pattern persisted across breeding stages (incubation: BG − CG = 28.0% dives < 5 km from the ice edge, *p* = 0.009, chick rearing: BG − CG = 13.8%, *p* < 0.001). Nevertheless, 73.6% of BG dives remained > 5 km from the ice. Several of these (Fig. S6) were within a fjord (Arnarfjӧrður), which was covered by the core foraging area (50% UD) of BG, but not of CG (Fig. 1B). BG foraged in fjords more frequently than CG (Fig. S6; number of foraging segments in a fjord (CG: 7%, BG: 34%) vs. not in a fjord, differed between species: χ^2^(df = 1, N = 610) = 35.5, *p* = 2.55e–9).

At Látrabjarg, dives within *CG\BG* were in waters of the same temperature as those in *CG ∩ BG* (Fig. S7, see “Niche overlap” section in “Methods” for notation definitions), while dives within *BG\CG* were in waters of different temperatures than dives in *CG ∩ BG*: warmer in fjords, and colder outside of fjords (Fig. S7). In contrast, at Grímsey, dives within *CG\BG*, *BG\CG* and *CG ∩ BG* were in waters of the same temperature (Fig. S7). These analyses suggest niche displacement of some BG foraging areas into distinct habitats from those occupied by CG at Látrabjarg, while spatial segregation at Grimsey occurred within the same habitats.

## Discussion

Our findings reveal stronger differences in habitat use at the site with the most varied available habitats but, contrary to our prediction, higher population sizes, driving higher competition levels, did not necessarily result in higher interspecific segregation.

We found that overall competition could be important in determining foraging ranges, but not spatial segregation among species. Birds from Látrabjarg ranged further from the colony than those from Grímsey (Fig. 1, Table S3). Foraging ranges are positively related to colony size in seabirds^34^ and the Látrabjarg colony is ~ 4.8 times larger than that at Grímsey^23^. From the likely stronger total competition at Látrabjarg, we expected species to be more spatially segregated there than at Grímsey. This would have been consistent with previous results: at a small colony in Greenland (~ 3000 pairs), no horizontal segregation was observed during the breeding season^35^, while at a larger colony in Labrador (~ 66,000 pairs^36^), horizontal segregation appeared^37^. In contrast, we found similar levels of spatial segregation, regardless of colony sizes, in these two Icelandic colonies.

Differences in dive depths among species were not evident at Látrabjarg and were weak at Grímsey. In contrast, studies at large colonies in the Bering Sea found evidence for vertical segregation^38,39^, but that at a small Greenland colony did not^35^, which is consistent with stronger competition producing greater segregation. Temporal segregation of dives was also only evident at Grímsey, with CG diving throughout the diel cycle and BG diving mainly in early and late hours. In the Bering Sea, similar temporal segregation was found at Bogoslof Island^39^ but not at St George Island, an extremely large colony^38^, suggesting that population size alone cannot explain patterns of temporal segregation. Diel patterns in seabird foraging activity are often related to foraging on prey performing diel vertical migration (DVM), so temporal segregation could be due to different prey preferences. However, diel activity patterns can arise from sex-specific parental-care strategies even at sites where DVM is likely limited^40^, and it is possible that unbalanced sampling across sexes could have created this apparent interspecific segregation (see Supporting Information).

At Látrabjarg during chick rearing, both species reduced their foraging ranges and spatial segregation ceased. Changes in interspecific spatial segregation with breeding stage, similar to that found among albatross species^41^, are due to trips becoming shorter to allow high chick provisioning rates. However, competition within this contracted foraging range is increased further as chicks’ food requirements increase resource demand^39^. We could therefore have expected segregation along an alternative axis to balance this, but this was not evident in dive depths or timings (Table 1). Segregation could instead be achieved through dietary divergence, as found for these species in the Pacific^39^. There, the evidence suggests that when resources are scarce, CG maintain diet composition, while their subordinate BG switch to lower quality generalist diets^5^.

At the site with the highest levels of competition, habitats are also more contrasted, and certain habitats might serve as refugia for BG against their apparently dominant CG competitors. Segregation in environmental space was strongest at Látrabjarg: BG regularly dived in colder waters than CG (Figs. 2 and S7) and travelled to the ice edge in the Denmark Strait—similar to earlier tracking findings from this same colony^42^ and consistent with BG foraging along the ice edge elsewhere^43^. These cold accessible habitats can be considered as refugia (sensu^12^), in the same way as, e.g. Swedish mountains, for Arctic char *Salvelinus alpinus* against more temperate competitors^44^. The availability of these refugia could, among many other factors**,** contribute to the much higher BG:CG ratio at Látrabjarg. In contrast, no marked interspecific difference was evident at Grímsey, where SST gradients were less pronounced, and from which the ice edge was inaccessible.

We also identified fjords as another potential refugium for BG from Látrabjarg, despite their warmer SSTs in the year of tracking. A propensity to fjord foraging was also evident for BG breeding in Svalbard^45^ and NW Greenland^46^. Here, only one fjord was used, despite others being easily accessible. Fjords vary in their characteristics and associated biophysical processes (bathymetric features, freshwater inflow, etc.). Arnarfjӧrður is deeper than the surrounding fjords, and contains submerged moraines^47^ which may enhance prey availability (see Supplementary Information).

The reliance of BG upon sea ice as a refugium could render them vulnerable to climate change that is causing Arctic sea ice extent to retreat^48^. Seabirds may switch forage habitats to respond to the retreat of sea ice away from their colony locations^49,50^. Alternatively, BG could forage further afield to track the ice edge, although this could mean reduced breeding success as provisioning rates decrease with increasing commuting times. In addition, sea ice loss could bring BG into closer competition with CG as the latter start to utilise areas previously unfavourable due to extensive sea ice. Fjords could represent another potential refugium when the ice retreats, but the climate effects on prey availability within fjords such as Arnarfjӧrður remain unknown, and the extent of this habitat is probably insufficient to support the entire BG population at Látrabjarg.

Both species have declined in Iceland^23^, possibly because of sudden regime shifts that have affected prey fields^51^. Nonetheless, BG tend to decline faster, particularly at southern colonies from which they almost disappeared^23^. Climate change, causing the loss of their habitat refugia, could amplify this trend by disproportionately affecting BG compared with CG at a site—Látrabjarg—that is currently still relatively stable and hosts ca. 1/3 of the Icelandic population^23^.

Overall, our study highlights the importance of foraging habitat refugia at sites where competition is high. When or where these refugia are absent or inaccessible, large proportions of the dominant species may overlap with their subordinate competitors. Further research could help understand whether the accessibility and carrying capacity of these refugia have demographic implications, with a special attention to these refugia that might be affected by climate change.

## Supplementary Information

Supplementary Information.

## Data Availability

All data was deposited at the UK Polar Data Centre. These will be made publicly available shortly at 10.5285/C7EC736C-E1B0-48B5-B9B9-E68AA5ADFCC3.
