# Supplementary material for: Effects of competitive pressure and habitat heterogeneity on niche partitioning between Arctic and boreal congeners
Source: Sci Rep. 2021 Nov 11;11:22133. doi: 10.1038/s41598-021-01506-w (PMC8586341; doi:10.1038/s41598-021-01506-w)
Supplement: Supplementary file 1 — Supplementary Information. [file 41598_2021_1506_MOESM1_ESM.pdf]

Supplementary materials

Supplementary methods

Deployment details

Breeding birds were captured at their nest sites using a noose pole. GPS loggers weighted ~6g and were attached dorsally on the feathers using a combination of Tesa® tape, glue and epoxy resin; TDR loggers weighted <2g and were attached ventrally on the feathers by the same method. The total device load represented <1% of the birds' weight, a threshold identified by Bodey et al. (2018) as limiting the effect of devices on birds' survival and reproduction. In both colonies, birds were tracked for a few days (0.6-11.4 days), as longer tracking durations were not possible, due to birds plucking off the loggers. GPS devices had a saltwater switch that delayed GPS reception when the device was submerged and triggered a haul out mode when the device had been dry for over one hour, saving power when the bird was underwater or at the colony. Devices were recovered upon recapture, after which biometrics were measured and blood and feather samples were collected for a separate set of analyses.

Behavioural classification

For each bird, time was separated into each of the following activities: at the colony, flying, on the sea surface and diving. This was done following a similar approach to Tremblay (2003) and Linnebjerg et al. (2014). Dives were identified as described in Methods. Time on the sea surface was characterised by constant and relatively cold recorded temperatures. Flying from the colony was characterised by decreasing then stabilising temperatures, whereas flying from the sea was characterised by generally increasing then stabilising temperatures. Finally, time at the colony was characterised by very variable and high temperatures. Distance from the

colony from the GPS data (see below) was used in conjunction with TDR data to confirm classifications.

Sea surface temperatures at diving locations

For each section identified as "on the sea surface", separated by segments of flight, sections corresponding to diving were removed and the average surface temperature recorded by the TDR was calculated. Dives were assigned to their corresponding "on the sea surface" period to analyse dive patterns in relation to SST.

Tests of interspecific overlap along each axis

Whether the two species overlapped less than by chance was tested following Geange et al. (2011), i.e. by permuting species labels across individuals and re-calculating the overlap indices, repeating the operation 1,000 times and estimating the pseudo-*p*-value as the proportion of simulated overlap values that are lower than the observed. A similar permutation method was used by [5] to test for the significance of overlap values derived from auk foraging data. At Látrabjarg, the same permutation approach was used to contrast the use of the ice edge by the two species, in this case contrasting the observed difference between BG and CG in proportions of dives < 5 km away from the ice edge, with its simulated equivalent.

Test of differences in overlap between sites (bootstrap approach)

Overlap values were compared between sites or breeding stages using a bootstrap procedure. Each iteration worked as follows: for each site (or breeding stage) and species, individuals were sampled with replacement, maintaining the observed sample size (e.g. 33 sampled CG at Látrabjarg) and calculated the overlap indices for each horizontal, vertical, temporal, and

habitat axis as described above. After 1000 iterations, for each axis, 95% confidence intervals (CI) were calculated using the 2.5th and 97.5th percentiles of the overlap indices at each site or breeding stage and of the differences between sites or breeding stages. If the 95% CI of the inter-site (or inter-stage) difference did not overlap with zero, the difference was considered as significant.

Supplementary discussion

Drivers of temporal segregation

Temporal segregation appeared between the studied BG and CG only at Grímsey. This segregation could be driven by different prey preferences, as diel activity patterns are usually related to foraging on prey performing diel vertical migration (DVM), as was found in both CG and BG at lower latitudes [6], [7]. However, Grímsey is far enough north to experience the midnight sun in midsummer and whether diel vertical migration continues in these conditions is debated [8], [9].

Alternatively, heterogeneous diel activity patterns can persist in BG even in the high Arctic in the form of sex-specific inverted rhythms [10]. If our sampling was biased toward one sex for this species at this site, these temporal segregation patterns might reflect more sexual segregation that interspecific segregation. Unfortunately, the sex of the tracked birds is unknown. Subtle morphological differences between the sexes exist [11], but the overlap between the sexes remains quite high and variations between colonies can be larger than within-colony variations between the sexes [11], preventing us from exploiting morphometric data to this end.

The distribution of deployment times at the two sites could suggest some possible bias towards one sex. Indeed, temporal segregation between the sexes in BG is often related to one sex

attending the colony mostly at night while the other attends the colony mostly during the day [10], [12]. Deployment times at Grímsey are more narrowly concentrated than at Látrabjarg (Fig. S8), which could suggest a potential bias towards one sex, but only molecular sexing could allow us to be sure.

Nevertheless, when repeating the analyses at Látrabjarg with only the subset of individuals that were captured during the same hours of the day as birds at Grímsey, the results did not change. Temporal overlap between BG and CG was 0.81, and was still not significantly lower than expected by chance (p-value = 0.138). If the temporal segregation observed at Grímsey was only due to biased sampling towards one sex, we would have expected temporal segregation to appear at Látrabjarg when biasing the sampling in the same way, but we did not observe this. This suggests that the sex of the birds is likely not the only factor responsible for the difference between the two sites.

Arnarfjörður

Arnarfjörður is a popular fjord for fisheries targeting the Northern shrimp *Pandalus borealis* [13], even though the stocks have recently declined [14]. The species is a known prey of Brünnich's guillemots in Svalbard [15], so BG from Látrabjarg could be targeting this species in Arnarfjörður. Note that another fjord accessible from Látrabjarg, Ísafjarðardjúp, is also known for its Northern shrimp stock [16]. However, conspecific seabirds at sea tend to segregate according to their colony of origin, and Ísafjarðardjúp is within foraging range of the very large colonies in the Hornstrandir [17], which could explain why Arnarfjörður is the only fjord used by birds from Látrabjarg.

Supplementary references

[1] T. W. Bodey *et al.*, “A phylogenetically controlled meta-analysis of biologging device effects on birds: Deleterious effects and a call for more standardized reporting of study data,” *Methods in Ecology and Evolution*, vol. 9, no. 4, pp. 946–955, 2018, doi: 10.1111/2041-210X.12934.

[2] Y. Tremblay, “Unconventional ventral attachment of time-depth recorders as a new method for investigating time budget and diving behaviour of seabirds,” *Journal of Experimental Biology*, vol. 206, no. 11, pp. 1929–1940, Jun. 2003, doi: 10.1242/jeb.00363.

[3] J. F. Linnebjerg, N. P. Huffeldt, K. Falk, F. R. Merkel, A. Mosbech, and M. Frederiksen, “Inferring seabird activity budgets from leg-mounted time–depth recorders,” *J Ornithol*, vol. 155, no. 1, pp. 301–306, Jan. 2014, doi: 10.1007/s10336-013-1015-7.

[4] S. W. Geange, S. Pledger, K. C. Burns, and J. S. Shima, “A unified analysis of niche overlap incorporating data of different types,” *Methods in Ecology and Evolution*, vol. 2, no. 2, pp. 175–184, 2011, doi: 10.1111/j.2041-210X.2010.00070.x.

[5] I. Pratte, G. Robertson, and M. Mallory, “Four sympatrically nesting auks show clear resource segregation in their foraging environment,” *Marine Ecology Progress Series*, vol. 572, pp. 243–254, May 2017, doi: 10.3354/meps12144.

[6] P. M. Regular, G. K. Davoren, A. Hedd, and W. A. Montevecchi, “Crepuscular foraging by a pursuit-diving seabird: tactics of common murres in response to the diel vertical migration of capelin,” *Marine Ecology Progress Series*, vol. 415, pp. 295–304, Sep. 2010, doi: 10.3354/meps08752.

[7] K. H. Elliott and A. J. Gaston, “Diel vertical migration of prey and light availability constrain foraging in an Arctic seabird,” *Mar Biol*, vol. 162, no. 9, pp. 1739–1748, Sep. 2015, doi: 10.1007/s00227-015-2701-1.

[8] F. R. Cottier, G. A. Tarling, A. Wold, and S. Falk-Petersen, “Unsynchronised and synchronised vertical migration of zooplankton in a high Arctic fjord,” *Limnology and Oceanography*, vol. 51, no. 6, pp. 2586–2599, 2006, doi: 10.4319/lo.2006.51.6.2586.

[9] K. Blachowiak-Samolyk *et al.*, “Arctic zooplankton do not perform diel vertical migration (DVM) during periods of midnight sun,” *Marine Ecology Progress Series*, vol. 308, pp. 101–116, Feb. 2006, doi: 10.3354/meps308101.

[10] N. P. Huffeldt and F. R. Merkel, “Sex-specific, inverted rhythms of breeding-site attendance in an Arctic seabird,” *Biol. Lett.*, vol. 12, no. 9, p. 20160289, Sep. 2016, doi: 10.1098/rsbl.2016.0289.

[11] R. A. Orben, R. Paredes, D. D. Roby, D. B. Irons, and S. A. Shaffer, “Body size affects individual winter foraging strategies of thick-billed murres in the Bering Sea,” *Journal of Animal Ecology*, vol. 84, no. 6, pp. 1589–1599, 2015, doi: https://doi.org/10.1111/1365-2656.12410.

[12] R. P. Paredes, I. L. J. L. Jones, D. J. B. J. Boness, Y. T. Tremblay, and M. R. Renner, “Sex-specific differences in diving behaviour of two sympatric Alcini species: thick-billed murres and razorbills,” *Canadian Journal of Zoology*, May 2008, doi: 10.1139/Z08-036.

[13] MFRI Assessment Reports., “Shrimp in Arnarfjörður – raekja í Arnarfjirði, *Pandalus borealis*.,” 2020.

[14] I. G. Jónsdóttir, H. Bakka, and B. T. Elvarsson, “Groundfish and invertebrate community shift in coastal areas off Iceland,” *Estuarine, Coastal and Shelf Science*, vol. 219, pp. 45–55, Apr. 2019, doi: 10.1016/j.ecss.2019.01.022.

[15] O. J. Lønne and G. W. Gabrielsen, “Summer diet of seabirds feeding in sea-ice-covered waters near Svalbard,” *Polar Biol*, vol. 12, no. 8, pp. 685–692, Dec. 1992, doi: 10.1007/BF00238868.

[16] MFRI Assessment Reports., “Shrimp in Ísafjarðardjú – raekja í Ísafjarðardjúpi, *Pandalus borealis*.,” 2020.

[17] A. Garðarsson, G. A. Guðmundsson, and K. Lilliendahl, “Svartfugl í íslenskum fuglabjörgum 2006-2008,” *Bliki*, vol. 33, pp. 35–46, 2019.

Supplementary figures

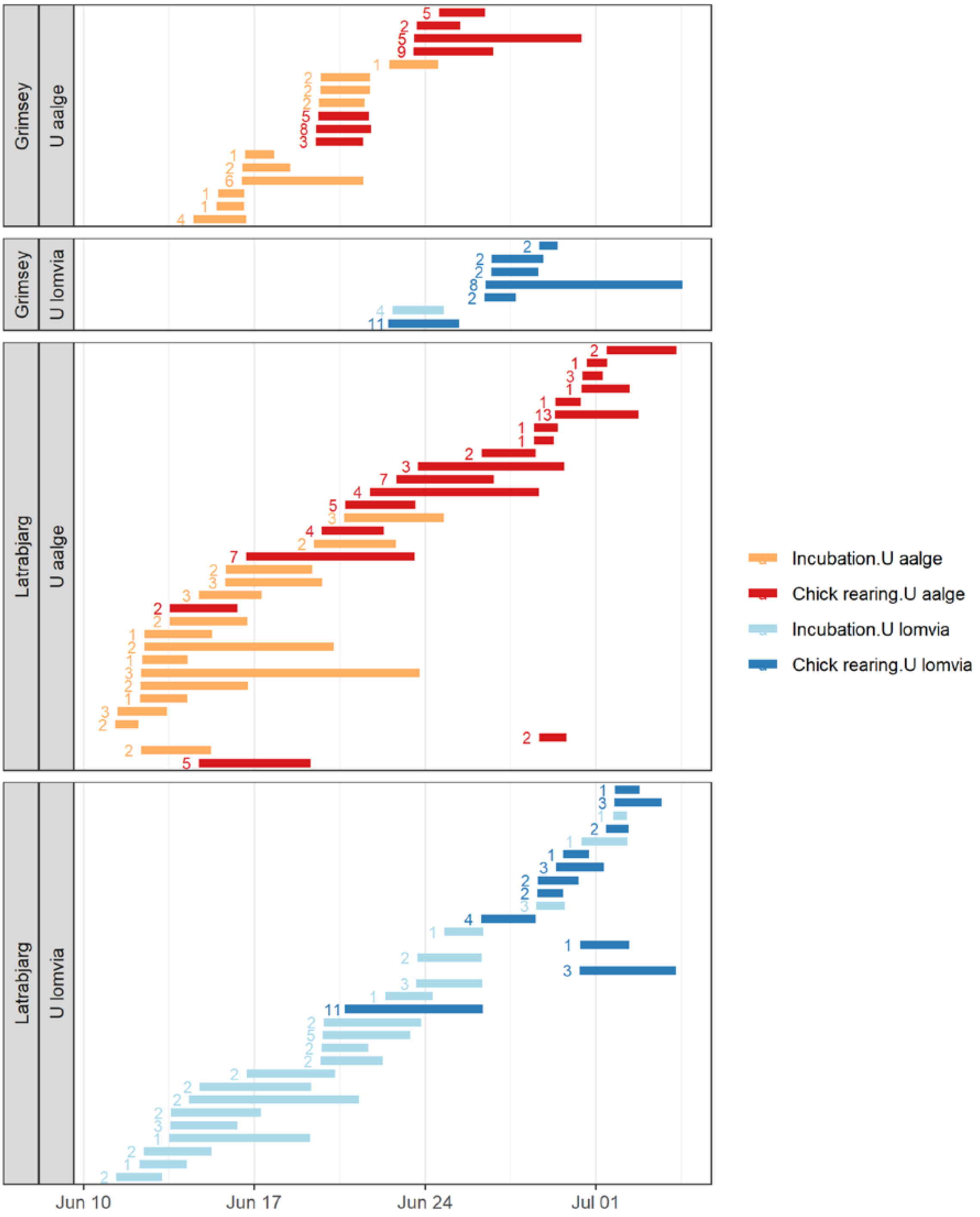

Figure S1. *Summary of tracking data. Lines represent the duration for which each individual was tracked. Orange and red: Common guillemots,* U. aalge*; Light and dark blue: Brünnich's guillemots,* U. lomvia. *Orange and light blue: tracking started during incubation.*

Red and dark blue: tracking started during chick rearing. Numbers indicate number of trips for each individual.

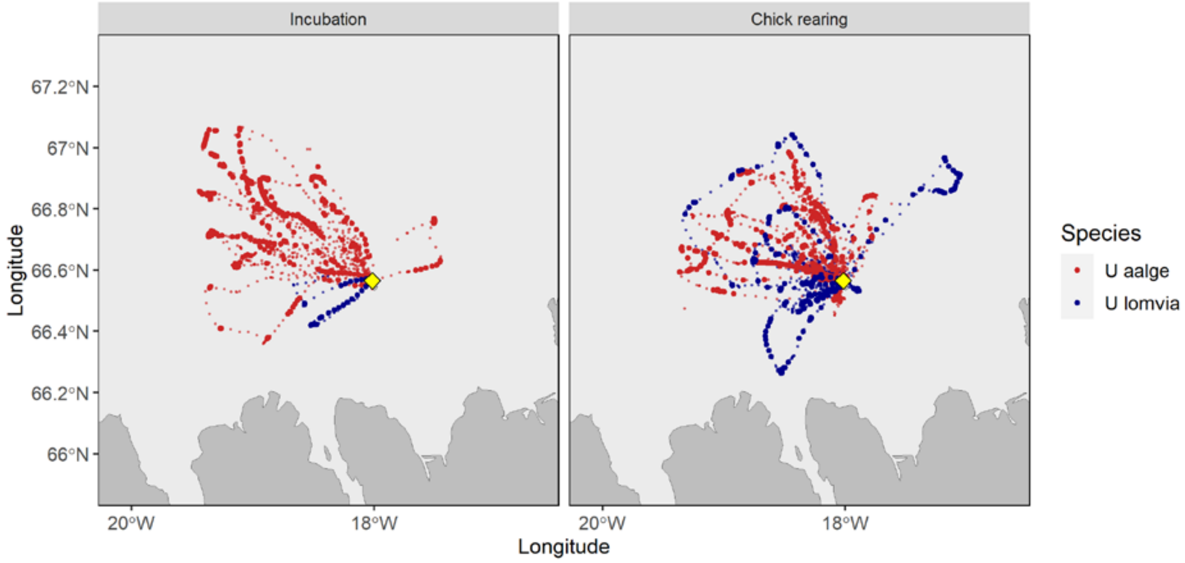

Figure S2. *Maps of the linearly-interpolated tracking data (small dots) and predicted dive locations (large dots) from Grímsey, separated by species (red: common guillemot,* U. aalge*; blue: Brünnich's guillemot,* U. lomvia*) and breeding stage. For both species combined, foraging locations were similar across breeding stages. Maps generated in R, version 4.1.1. (https://www.R-project.org/).*

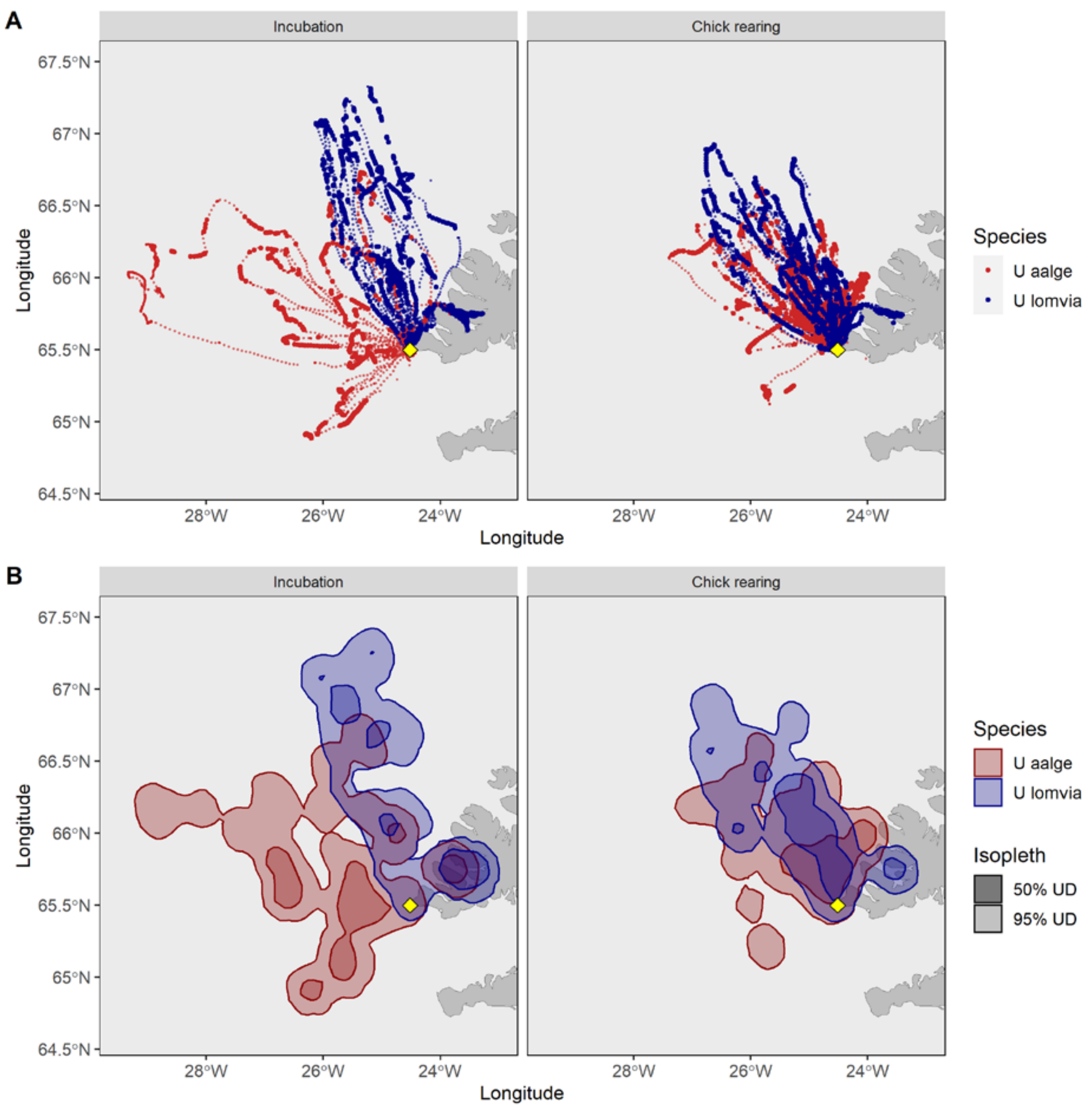

Figure S3. A. *Maps of the linearly-interpolated tracking data (small dots) and predicted dive locations (large dots) from Látrabjarg, separated by species (red: common guillemot,* U. aalge*; blue: Brünnich's guillemot,* U. lomvia*) and breeding stage.* ***B.*** *At-sea utilisation distributions (UD) and their overlap. Red: common guillemot 50% UD (dark) and 95% UD (pale); blue: Brünnich's guillemot 50% UD (dark) and 95% UD (pale). Bandwidth used for kernel calculations: 7.5km. Maps generated in R, version 4.1.1. (https://www.R-project.org/).*

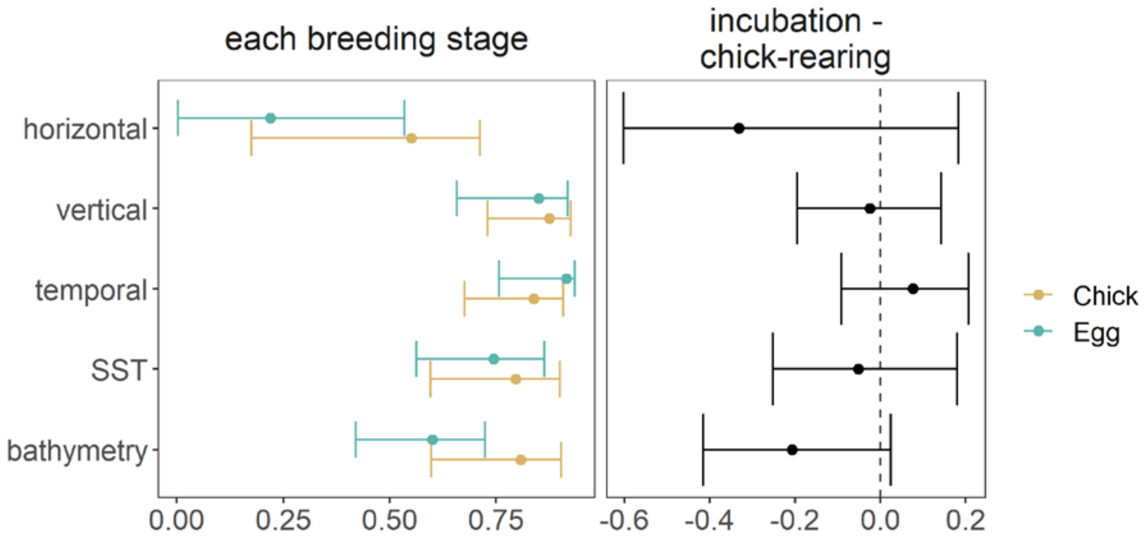

Figure S4. *Overlap values for each breeding stage (left) and difference between incubation and chick rearing (right), at Látrabjarg only: observed values and bootstrap confidence intervals. Positive values in the right panel correspond to axes along which the overlap is higher during incubation than chick rearing (i.e. species are more segregated during chick rearing). SST = Sea Surface Temperature.*

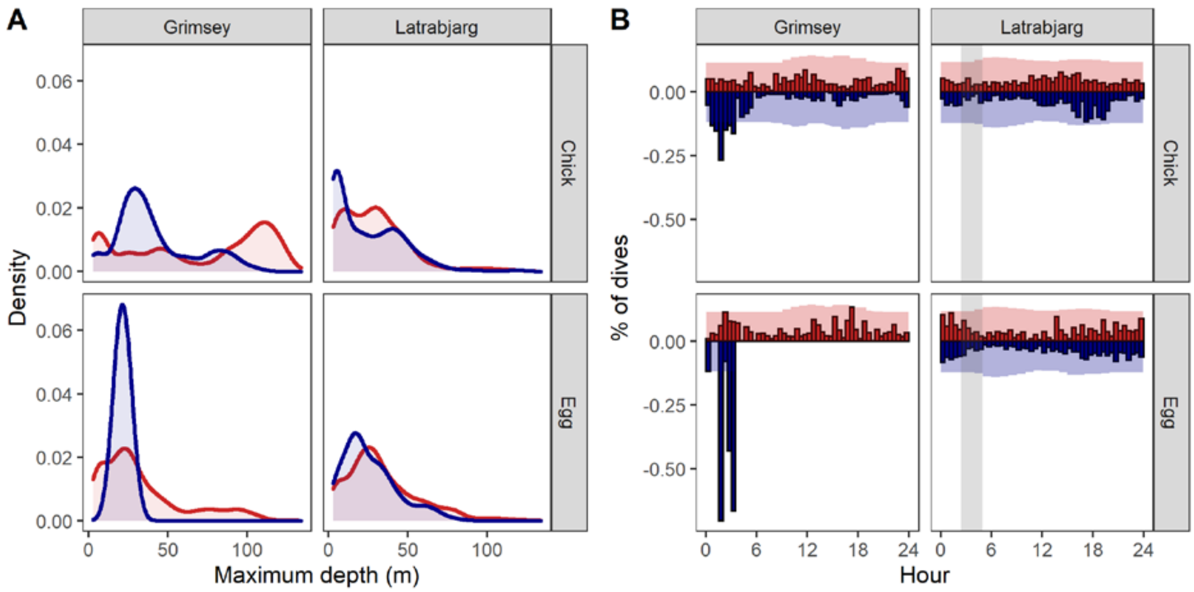

***Figure S5. Diving behaviour across colonies, species and breeding stages** (red: common guillemot; blue: Brünnich's guillemot). A: Distribution of maximum dive depth; B: Diel distribution of the occurrence of dives; shaded areas: relative distribution of tracking effort throughout the day (hours in GMT); grey areas indicate times between sunset and sunrise (averaged over all deployment dates for each site);*

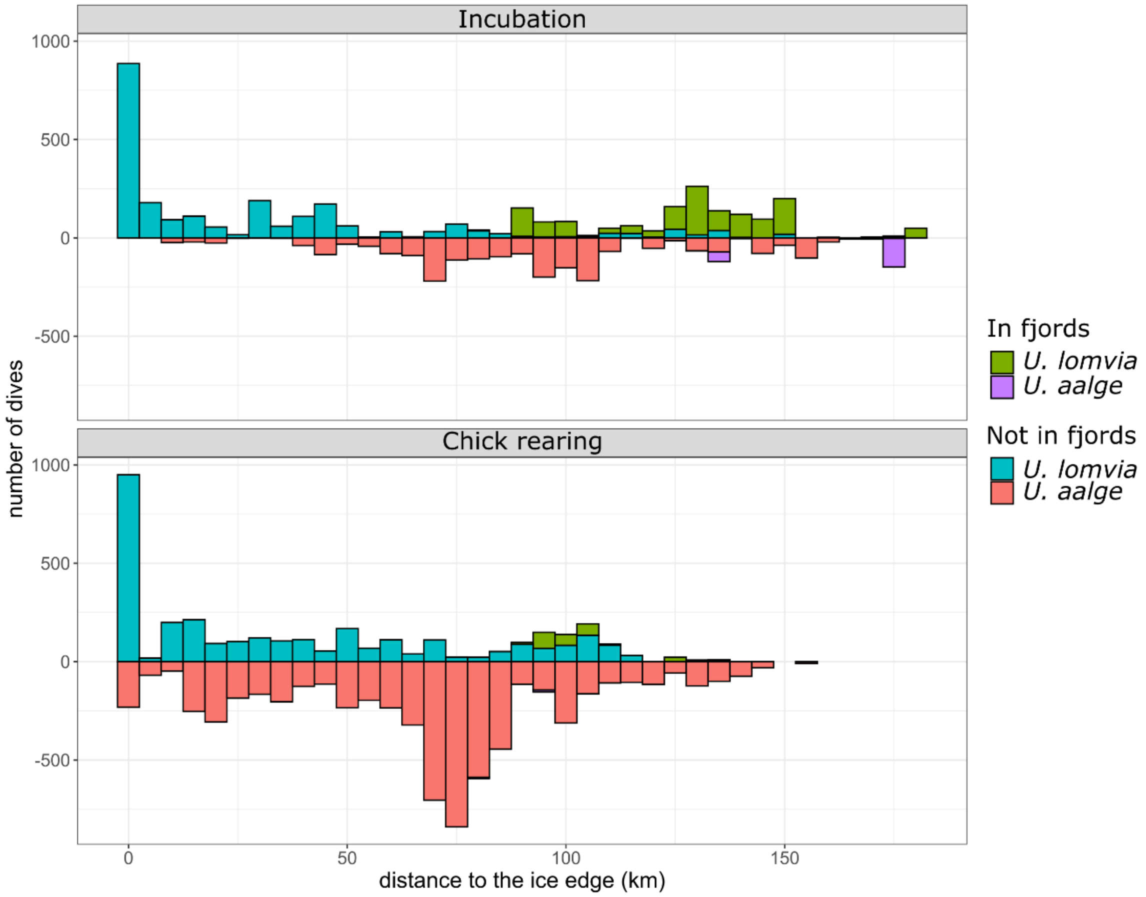

Figure S6. *Minimum distance between dive locations and the ice edge the previous day, for Brünnich's guillemot (green: in fjords, blue: outside of fjords) and common guillemot (purple: in fjords, salmon: outside of fjords) breeding at Látrabjarg, contrasted between incubation and chick rearing.*

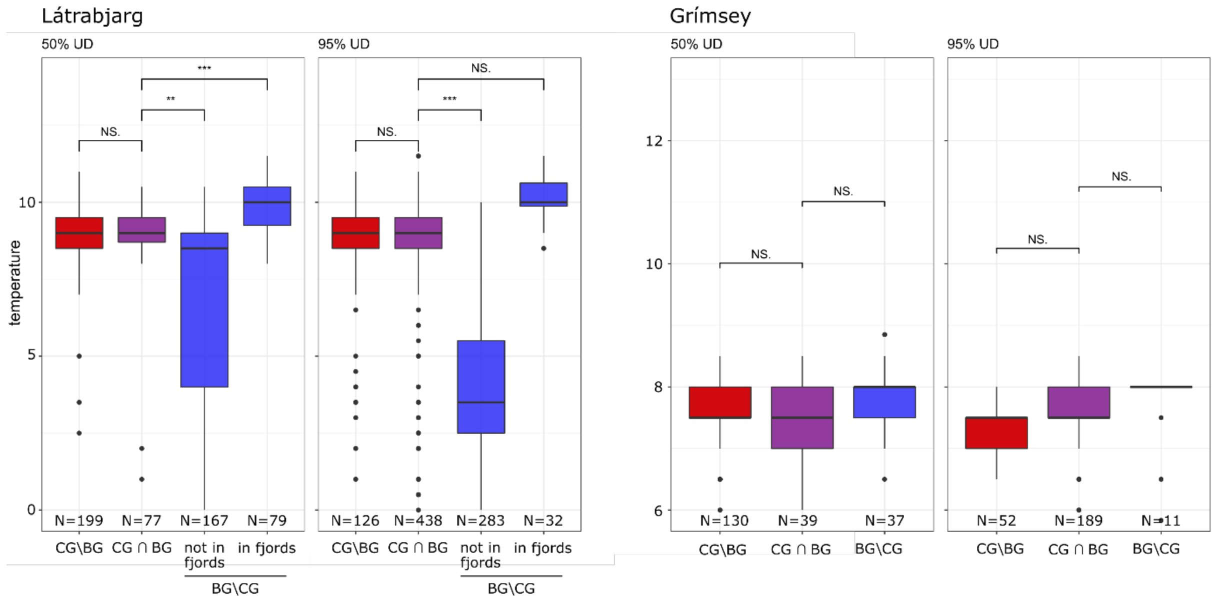

Figure S7. *Temperatures around Látrabjarg and Grímsey: temperature (in ˚C) around diving sections (irrespective of species) falling into CG ∩ BG, the overlapping area of the common (CG) and Brünnich's guillemots (BG) utilisation distributions (UDs), into CG\BG, the part of the CG UD that was outside of CG ∩ BG, or into BG\CG, the part of the BG UD that was outside of CG ∩ BG. The latter was separated into dives that were in fjords and dives that were not. Results presented for the 50% UDs (left) and the 95% UDs (right). Significance levels (NS. = non-significant, ** = 'p-value < 0.01', *** = 'p-value < 0.001') correspond to results of GLMMs with temperature as a response variable, location category as a fixed effect, and trip nested within individual as random effects.*

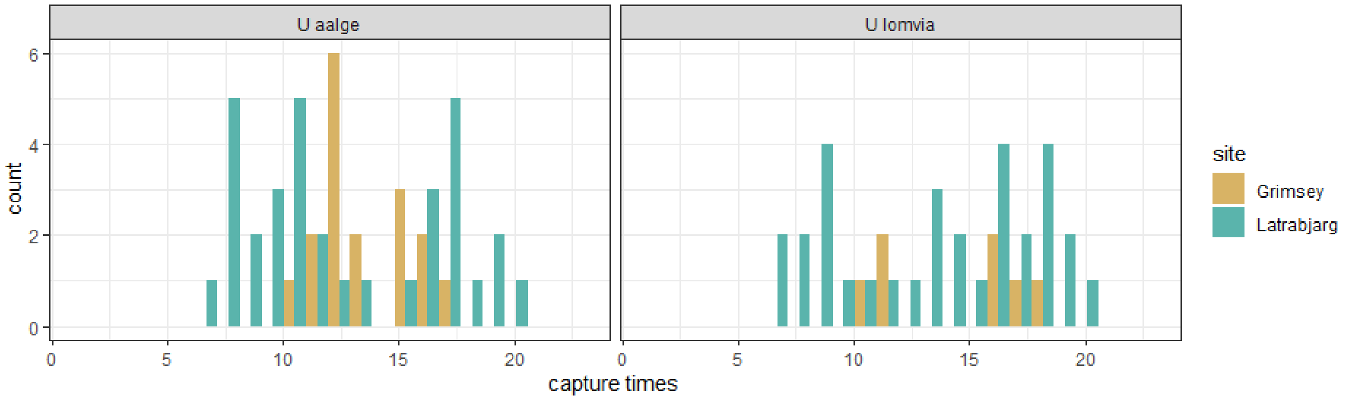

Figure S8. *Distributions of capture times (hour of the day) of the tracked birds, for each species at each site.*

Supplementary tables

Activity	Diving	At sea not diving	Flying	At the colony
Depth	<3m	-	-	-
Temperature	Varying in conjunction with depth (typically cold)	Constant and relatively cold; varies with water masses visited	Increasing then stabilising, gets relatively warm	Very variable and high
Distance to the colony	Constant (or slowly drifting)	Constant (or slowly drifting)	Changing quickly	= 0

Table S1. *Splitting of activities using combined TDR and GPS data.*

Site	Species	Breeding stage	GPS and TDR deployed	Recap-tured birds	GPS retrieved	TDR retrieved	Birds in analyses
Látrabjarg	*U. aalge*	Incubation	20	15	15	15	15
		Chick-rearing	18	18	18	18	18
	U. lomvia	Incubation	23	20	20	20	20
		Chick-rearing	12	11	11	11	11
Grímsey	*U. aalge*	Incubation	17	13	12	12*	10
		Chick-rearing	8	7	7	7	7
	U. lomvia	Incubation	3	2	1	2	1
		Chick-rearing	10	7	6	7	6
		**Total**	**111**	**93**	**90**	**92***	**88**

Table S2. *Sample sizes of GPS and TDR deployments and retrievals, and number of birds used in the analyses. Only birds for which both GPS and TDR data were used in the analyses. *Note that, at Grímsey, one bird for which both the GPS and the TDR could be retrieved could not be used in the analyses because of a malfunctioning TDR. When the GPS or TDR logger could not be retrieved from a recaptured bird, it was because the logger was lost due to birds plucking it off.*

	Estimate ± SE		
	**Log(maximum distance in km)**	**Log(trip duration in h)**	**Log(Duration of flying sections in min)**
Grimsey	3.08 ± 0.18	1.59 ± 0.15	2.05 ± 0.09
Difference between Látrabjarg and Grimsey	0.52 ± 0.22	0.46 ± 0.18	0.37 ± 0.11

Table S3. *Parameter estimates from minimum adequate LMMs with log-transformed trip duration, trip maximum distance from the colony and duration of flight sections as response variables, site, species and stage selected from as fixed effects and individuals as random effects. Coefficient estimate ± SE for each variable retained in the final model (for details of model selection, see Table S4). Incomplete trips (i.e. with large gaps in GPS data at the start or end of the trip) were excluded to calculate these values.*

	(Intercept)	site	species	stage	site: species	site:stage	species: stage	site:stage :species	ΔAICc
**Trip maximum distance**	3.08	**X**							0.00
	2.97	X		X		X			1.99
	3.44								2.08
	3.16	X		X					2.33
	3.08	X	X						3.40
	2.97	X	X	X	X	X	X	X	3.43
	3.21	X	X		X				3.54
	3.21	X	X	X	X		X		4.13
	3.52			X					4.88
	3.35	X	X	X	X				5.01
	3.08	X	X	X	X	X	X		5.11
	2.94	X	X	X		X			5.19
	3.14	X	X	X	X	X			5.20
	3.41		X						5.27
	3.16	X	X	X					5.73
	3.02	X	X	X			X		6.12
	2.85	X	X	X		X	X		6.19
	3.49		X	X					8.03
	3.43		X	X			X		9.47
**Trip duration**	1.59	**X**							0.00
	1.46	X		X					0.28
	1.74			X					1.85
	1.92								2.47
	1.46	X		X		X			2.53
	1.61	X	X						3.61
	1.49	X	X	X					3.83
	1.68	X	X		X				5.06
	1.75		X	X					5.57
	1.43	X	X	X			X		5.65
	1.54	X	X	X	X				5.79
	1.50	X	X	X		X			6.04
	1.92		X						6.16
	1.41	X	X	X	X	X	X	X	6.97
	1.48	X	X	X	X		X		7.37
	1.45	X	X	X		X	X		7.78
	1.74		X	X			X		7.87
	1.56	X	X	X	X	X			7.96
	1.53	X	X	X	X	X	X		9.37
**Flight sections duration**	2.12	**X**	**X**						0.00
	2.05	X							1.22
	2.10	X	X		X				3.06
	2.15	X	X	X					4.11
	2.09	X		X					5.10
	1.97	X		X		X			5.18
	2.06	X	X	X		X			5.69
	2.11	X	X	X			X		7.07

2.14	X	X	X	X				7.21
2.31								7.53
2.03	X	X	X	X	X			8.59
2.04	X	X	X		X	X		8.92
2.38		X						9.63
2.11	X	X	X	X		X		10.13
2.34			X					11.79
2.02	X	X	X	X	X	X		11.81
2.02	X	X	X	X	X	X	X	13.11
2.41		X	X					14.03
2.42		X	X			X		17.25

Table S4. *Model ranking using AIC corrected for small sample sizes (AICc). Full models: LMMs with log-transformed trip duration, trip maximum distance from the colony and duration of flight sections as response variables, site, species and stage as fixed effects and individuals as random effects. ΔAICc calculated using the top ranked model as a reference.*

		Trip statistics			Flight bout statistics	
**Site**	**Species**	**Distance, in km (mean ± SE)**	**Duration, in hours (mean ± SE)**	**Number of trips (number of birds)**	**Duration, in min (mean ± SE)**	**Number of bouts (number of birds)**
Grímsey	*U. aalge*	30.8 ± 2.83	6.47 ± 0.614	59 (17)	12.4 ± 0.0401	321 (17)
	U. lomvia	19.7 ± 2.80	4.84 ± 0.644	31 (7)	9.25 ± 0.0674	138 (7)
Látrabjarg	*U. aalge*	51.6 ± 4.41	11.6 ± 1.65	96 (33)	20.4 ± 0.0439	494 (32)
	U. lomvia	67.0 ± 6.66	10.2 ± 0.925	73 (31)	21.4 ± 0.117	562 (28)

Table S5. *Summary trip and flight bout statistics. Standard errors (SE) calculated as standard deviation divided by total number of trips (all birds combined) for trip statistics, and standard deviation divided by total number flying bouts identified (all birds combined) for flight bout duration.*
